# Role of Dietary Metabolites in Regulating the Host Immune Response in Gastrointestinal Disease

**DOI:** 10.3389/fimmu.2017.00051

**Published:** 2017-01-27

**Authors:** Mohamad El-Zaatari, John Y. Kao

**Affiliations:** ^1^Division of Gastroenterology, Department of Internal Medicine-Gastroenterology, University of Michigan, Ann Arbor, MI, USA

**Keywords:** S1pr1, MDSC, CD8, cytotoxic, stomach, gastric, metaplasia, colorectal cancer

## Abstract

The host immune response to gastrointestinal (GI) infections, hypersensitivity reactions, or GI cancers comprises numerous pathways that elicit responses on different host cells. Some of these include (1) the stimulation of mast cells *via* their IgE receptor, (2) the production of antibodies leading to antibody-mediated cytotoxic T/natural killer cell killing, (3) the activation of the complement pathway, and (4) the activation of the adaptive immune response *via* antigen-presenting cell, T cell, and B cell interactions. Within the plethora of these different responses, several host immune cells represent major key players such as those of myeloid lineage (including neutrophils, macrophages, myeloid-derived suppressor cells) or lymphoid lineage (including T and B cells). In this review, we focus on newly identified metabolites and metabolite receptors that are expressed by either myeloid or lymphoid lineages. Irrespective of their source, these metabolites can in certain instances elicit responses on a wide range of cell types. The myeloid-expressed metabolic enzymes and receptors which we will discuss in this review include arginase 2 (Arg2), indoleamine-2,3-dioxygenase 1 (IDO1), hydroxycarboxylic acid receptor 2 (Hcar2; also called GPR109A), and immunoresponsive gene 1 (Irg1). We will also review the role of the lymphoid-expressed metabolite receptor that binds to the sphingosine-1-phosphate (S1P) sphingolipid. Moreover, we will describe the synthesis and metabolism of retinoic acid, and its effect on T cell activation. The review will then discuss the function of these metabolites in the context of GI disease. The review provides evidence that metabolic pathways operate in a disease- and context-dependent manner—either independently or concomitantly—in the GI tract. Therefore, an integrated approach and combinatorial analyses are necessary to devise new therapeutic strategies that can synergistically improve prognoses.

## Introduction

The host immune response—during infections or carcinogenesis—comprises a plethora of stimulatory and inhibitory signals. These manifest in numerous cellular activities, which are underscored by extensively complicated, variegated, and overlapping molecular and cellular interactions. Metabolites—such as those generated by amino acid breakdown—comprise a subset of the above-mentioned signals, which can regulate the outcome of host immunopathology in a context-dependent (i.e., disease-specific) manner.

In this article, we will first briefly review several components of the host immune response in the gastrointestinal (GI) tract. Then, we will outline specific contexts in which metabolites and/or metabolite receptors influence these components of host immunity. We will describe the source of these metabolites, and the critical steps that can control the immunopathological outcome. By contrast, we will also discuss the manner in which failed approaches can arise in targeting these pathways. Due to our inability to review the entire metabolome, we will focus on eight specific metabolites and their receptors, which epitomize distinct and variegated responses. The review will focus on the roles of arginase 2 (Arg2), indoleamine-2,3-dioxygenase 1 (IDO1), hydroxycarboxylic acid receptor 2 (Hcar2; also called GPR109A), immunoresponsive gene 1 (Irg1), sphingosine-1-phosphate (S1P), and all-trans-retinoic acid (atRA).

## Host Immunity in the GI Tract

The host immune response comprises the initiation of several reactions that are carried out by a subset of white blood cells (WBCs), or leukocytes. These WBCs are divided into two lineages, myeloid and lymphoid, which arise from a hematopoietic stem cell origin (1). The hematopoietic stem cell gives rise to common lymphoid progenitors (CLPs) and common myeloid progenitors (CMPs) (1). The lymphoid lineage arises from the CLP and comprises B cells (and mature plasma cells), T cells, and natural killer (NK) cells (2, 3). The myeloid lineage arises from the CMP and comprises monocytes, macrophages, dendritic cells, neutrophils, eosinophils, basophils, mast cells, megakaryocytes, and erythrocytes (2, 3).

After a foreign antigen is detected by a subset of myeloid cells (4) or B cells (5), these cells can either elicit an autonomous thymus-independent response (6), or otherwise interact with T cells to elicit an acquired T cell-dependent response (6). The cells that detect the antigen are known as antigen-presenting cells (APCs) because they are able to present antigen to T cells (7). As mentioned above, APCs are usually myeloid (mainly dendritic cells) (4), but can also be of the B cell lineage (5). These cells detect foreign antigen in the mucosa of the affected GI tissue (8). However, they can also detect antigen in tissue-draining lymph nodes if the foreign antigen was in circulation and reached the lymph nodes *via* afferent lymphatic vessels (9–11). Once APCs detect foreign antigen they can elicit an autonomous T cell-independent response; for example dendritic cells can produce cytokines (12) while B cells can produce antibody in a T cell-independent manner (13). Alternatively, APCs can travel to the lymph nodes *via* afferent lymphatic ducts (12, 14) and interact with T cell receptors on T cells *via* their major histocompatibility II (MHC-II) molecules, and as a result elicit a T cell-dependent response (15, 16). The APC:T cell interaction usually occurs in the paracortex of tissue-draining lymph nodes (16), although this interaction can also occur in tertiary lymphoid organs of the affected tissue *in situ* (17–19). Antigen presentation by dendritic cells to T cells sequentially leads to T cell–B cell interactions, thus triggering B cell maturation and antibody production (20, 21).

Numerous variegated—but coincidental—cellular activities regulate the outcome of host immunity in the GI tract, but some of the pathways are epitomized by the activities observed during hypersensitivity reactions (22–24). In type I hypersensitivity, a target antigen leads to the stimulation of mast cells *via* their IgE receptors leading to degranulation (22–24). In type II hypersensitivity, antibodies bind to target cells leading to direct cell to cell killing by NK or CD8^+^ T cells (22–24). Type III hypersensitivity comprises the binding of complement to the target antigen leading to chemotaxis and infiltration of neutrophils (22–24). Type IV hypersensitivity is mediated by cytokine release from helper T cells that stimulate macrophage or killer T cell activity against target cells (22–24). Overall, these pathways do not only operate in hypersensitivity or autoimmune reactions, but also in regular pathogenesis (25) in response to infection or carcinogenesis. For example, cytokines that exacerbate host immunopathology are induced in the cecum during *Clostridium difficile* infection (26) and in the stomach during chronic *Helicobacter felis*-induced gastritis (27). Both of these pathological phenomena are reminiscent of type IV autoimmunity (22–24). In addition, autoantibody production (28–38) and complement activation (39, 40) are detected during *Helicobacter pylori* gastritis, which are also reminiscent of type II and type III autoimmunity, respectively (22–24).

In summary, this paragraph outlines some of the complexity of host immunity and its coincidental activities of targeting pathogen versus host. The complexity of these pathways renders it unattainable to review all these processes. Hence, we will focus our review on two processes for which metabolite involvement has been characterized to a certain extent: (1) the role of metabolites in regulating myeloid cell function (namely the roles of Arg2, IDO1, Hcar2, and Irg1), and (2) the role of metabolites that exert a direct effect on CD8^+^ T cell migration and memory (namely S1P), or on CD8^+^ T cell activation *via* epithelial cell mediation (retinoic acid).

## Arg2 in Suppressing Macrophage Cytotoxicity and Myeloid-Derived Suppressor Cell (MDSC) Function

**Figure 1:**
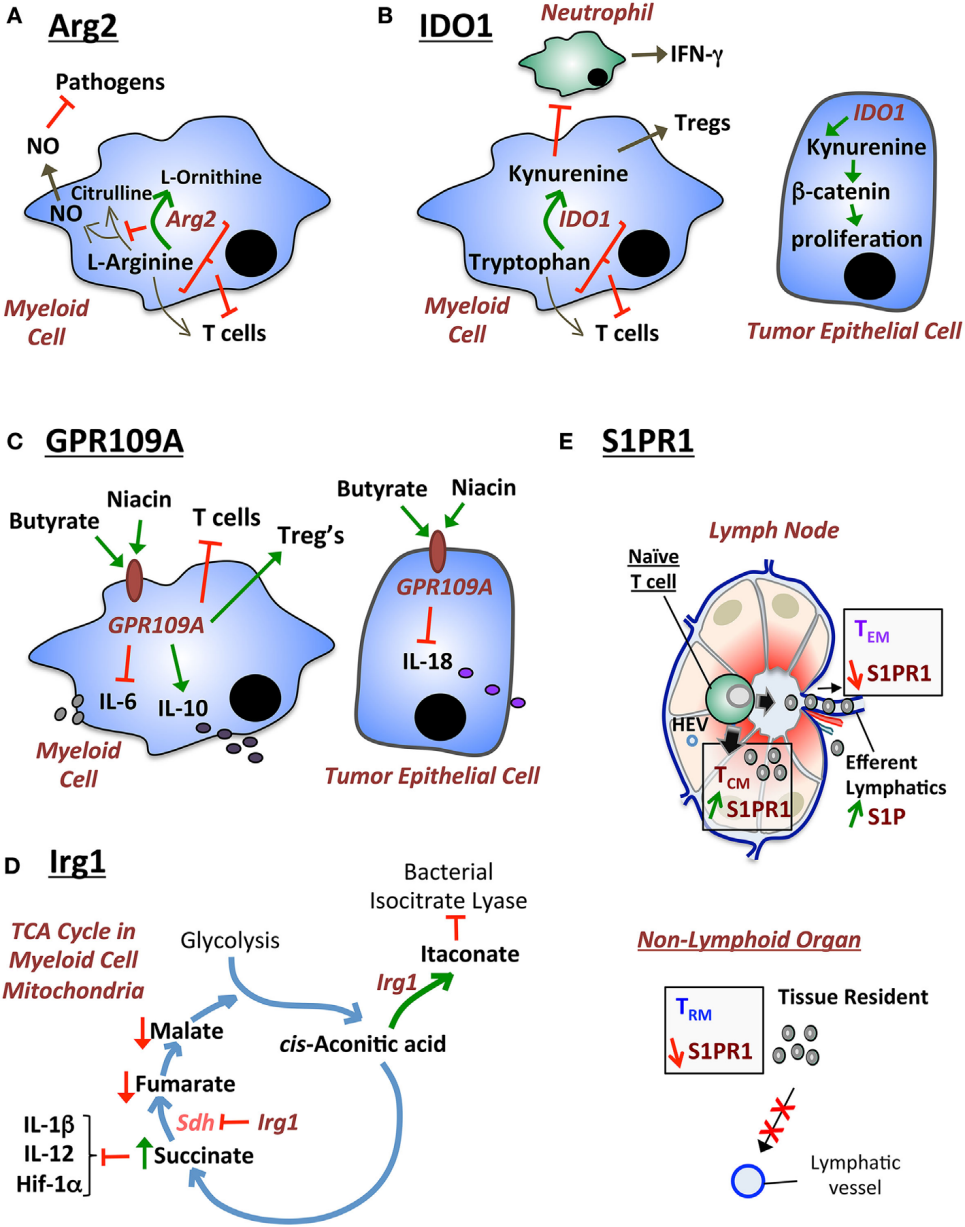
**Diagrammatical modeling of exemplary metabolic pathways that regulate host immunity**. **(A)** Arginase 2 (Arg2) functions by regulating two pathways: (i) depletion of l-arginine required for NO synthesis contributing to macrophage cytotoxic activity against pathogens; and (ii) depletion of l-arginine required for T cell immunity. **(B)** IDO1 has been traditionally described to suppress T cell immunity by depleting tryptophan. Moreover, IDO1 generates kynurenine that stimulates Tregs. However, new alternative mechanisms for IDO1 have recently been described: (i) IDO1 suppresses (potentially *via* kynurenine) IFN-γ-producing cecal neutrophils during *Clostridium difficile* colitis; and (ii) IDO1-produced metabolites (such as kynurenine) stimulate the β-catenin pathway and tumor epithelial cell proliferation in colorectal cancer. **(C)** GPR109A (also known as hydroxycarboxylic acid receptor 2) is the niacin/butyrate receptor which exhibits two previously described functions: (i) in myeloid cells GPR109A suppresses IL-6 and T cell immunity, while promoting IL-10 production and Treg differentiation; and (ii) in epithelial cells GPR109A suppresses IL-18 production. **(D)** Irg1 is an enzyme that regulates the tricarboxylic acid (TCA) (citric acid) cycle in the mitochondria of myeloid cells. Irg1 regulates two functions: (i) Irg1 generates itaconate, which exhibits antimicrobial activities by inhibiting the bacterial enzyme isocitrate lyase; and (ii) Irg1 inhibits succinate dehydrogenase (Sdh), which leads to an increase in succinate levels. Increased succinate suppresses pro-inflammatory cytokines (IL-1β, IL-12) and hypoxia-inducible factor 1 alpha (HIF-1α). **(E)** S1P provides a high gradient in efferent lymphatic vessels of the lymph node, which leads to the egress of memory T cells expressing high levels of the S1pr1 receptor. In non-lymphoid tissue, low levels of S1pr1 are necessary for sustaining tissue-resident memory T (T_RM_) cells by continuously preventing egress out of the tissue *via* lymphatic ducts.

Arginase 2 is an enzyme for which two previously defined mechanisms have been described: (1) Arg2 reduces macrophage cytotoxic activity (41) by depleting l-arginine availability for the production of nitric oxide (NO) by nitric oxide synthase (NOS) (42); and (2) Arg2 in MDSCs inhibits T cell immunity by depleting l-arginine availability for T cells in the inflamed microenvironment (43) (Figure 1A). l-arginine is a non-essential amino acid that is sufficiently produced by the human body, but becomes essential in disease contexts that upregulate Arg2 (44). Therefore, the use of l-arginine as a dietary supplement for treating disease is arguable since the pathway is regulated at the level of l-arginine breakdown. However, the metabolic enzyme Arg2 presents an attractive therapeutic target for modulating T cell immunity (45). In order to review the role of Arg2, it is important to outline the nature and function of MDSCs in tumor biology and autoimmune pathology.

Myeloid-derived suppressor cells are a myeloid population (that can be regulated by intrinsic Arg2 activity), which exerts immunosuppressive activities against T cells (46). The regulation of effector T cell function by MDSCs is critical in pathological situations (47, 48). The reason T cells are important is because, for example in cancer, CD8^+^ cytotoxic T cells exhibit antitumor activity by killing tumor cells (49). In viral infections, CD8^+^ T cells confer protection by eliminating virus-infected cells (49). However, the effect of MDSC on T cell function is also complicated by the fact that T cells exhibit variable phenotypes depending on two intertwined phenomena: (1) homing receptor expression, and (2) differentiation into specific T cell memory subtypes (50). The latter point will not be discussed here, but will be discussed in the last section of this article regarding S1P. Nevertheless, MDSCs are believed to play an unfavorable role in cancer and viral infections by suppressing T cell immunity (46). Hence, this suppressive activity of MDSCs against T cells is partly mediated by Arg2, which unravels an attractive therapeutic target against cancer (46). However, *in vivo* pathological situations warn of arguable outcomes for this strategy as will be described in the following paragraph.

The reason why the usefulness of Arg2 inhibitors against GI cancer is arguable is epitomized by a study in which Arg2 deficiency exacerbated gastric immunopathology during chronic gastritis (51, 52). The authors showed that this effect was not mediated by NOS (52). Even though the authors did not investigate the effect of reduced MDSC function on CD8^+^ T cell responses in this model, they did report a dramatic increase in tissue IFN-γ levels—a cytokine that is mainly produced by CD8^+^ T cells and NK cells (51, 52). Hence, if Arg2^−/−^ leads to reduced MDSC function and increased CD8^+^ T cell response—as reported in the literature—the worsened immunopathology in Arg2^−/−^ would suggest that CD8^+^ T cells are involved in an autoimmune etiology in the stomach. In this scenario, the use of Arg2 inhibitors to treat cancers becomes arguable since the inflamed normal tissue—adjacent to the tumor—might develop worsened immunopathology when Arg2 is inhibited. Therefore, one has to consider the unfavorable pathological outcome of Arg2 suppression, which might arise due to heightened inflammation in normal adjacent tissue to the tumor, which can exacerbate the development of preneoplastic metaplastic lesions.

## An Unusual Role for IDO1 in Suppressing Cecal IFN-γ-Producing Neutrophils

IDO1 is a tryptophan-catabolizing enzyme that suppresses T cell immunity (53), but has recently been shown to play major alternative roles (other than T cell regulation) in the GI tract (26, 54) (Figure 1B). The role of IDO1 in regulating T cell immunity was first described in the prevention of allogeneic fetal rejection (53). In the latter study, IDO1 was proposed to deplete tryptophan pools for T cells by catabolizing tryptophan into kynurenine (53). In correlation with this finding, IDO1 inhibitor increased CD8^+^ T cells and reduced tumor growth in a transgenic mouse model of cecal gastrointestinal stromal tumors, which was reversed by CD8^+^ T cell depletion (55). Furthermore, IDO1 is expressed by MDSCs and regulates the immunosuppressive function of these cells against T cells (56). Alternatively, the mechanism of anti-T cell immunity of IDO1 can also be attributed to plasmocytoid dendritic cell expression of IDO1 in tumor-draining lymph nodes (57). In contrast to the latter observations, our recent finding demonstrates that IDO1 plays other distinct functions in non-tumor inflammatory environments that do not employ T cell immunity as a major component (26). Moreover, another recent study also showed that IDO1 plays a critical role in regulating epithelial cell function, which affects the outcome of colorectal cancer development in mouse models (54). The latter two observations indicate that the functions of IDO1 are not limited to regulating T cell immunity, but are subject to variability based on pathogenic context. These two studies will be reviewed in the following two paragraphs.

In our recent study, IDO1 suppressed IFN-γ-producing neutrophils in *C. difficile* colitis, but had no apparent effect on cecal CD4^+^ T cell number (26). It is important to note that *C. difficile* colitis is an acute neutrophilic disease (58), in which T cells do not affect immunopathology or survival in a significant manner (59). The latter observation was supported by the use of Rag1^−/−^ mice, which succumbed to similar immunopathology relative to wild-type mice during *C. difficile* infection (59). Our observation that IDO1 deficiency led to increased cecal neutrophils (26) correlates with a recent finding that the IDO1 metabolite, kynurenine, suppresses neutrophil chemotaxis potentially *via* the aryl hydrocarbon receptor (60). The other surprising finding in our study was that cecal neutrophils—regulated by IDO1—were the major source (>90%) of IFN-γ (26). The latter observation also correlates with neutrophils being the major source of IFN-γ during *Salmonella enterica* Typhimurium-induced colitis (61). Therefore, the breakdown of the amino acid tryptophan into kynurenine in this context inhibits chemotaxis of IFN-γ-producing neutrophils during infectious colitis of the lower bowel. These findings unravel a novel role for IDO1 during acute infections of the GI tract, which warn of unfavorable side effects for IDO1 inhibitors. This is of special concern since cancer patients are more likely to succumb to *C. difficile* infections (62). Moreover, the findings also propose that IDO1 might potentially play alternative functions that regulate myeloid cells (e.g., neutrophils or MDSCs) in other contexts, such as tumor context. In support of the latter proposition, a recent paper showed that MDSCs were increased in IDO1-overexpressing B16 melanoma tumor models (63).

In addition to our observed effect of IDO1 on neutrophils, another study demonstrated a novel function of IDO1 in regulating epithelial cancer cell proliferation. The authors showed that IDO1 deficiency led to reduced tumor burden in the azoxymethane (AOM) and dextran sodium sulfate (DSS) model, which was mediated by a T cell-independent mechanism (54). The finding was similarly replicated in Rag1^−/−^ mice that lack T and B lymphocytes, thus corroborating that T cells were not involved (54). The authors proposed that IDO1 influenced tumor development by regulating epithelial cell proliferation *via* β-catenin (54). Therefore, we conclude—based on the data from the last two paragraphs—that IDO1 plays additional T cell-independent roles, which are variable according to disease context. Hence, these pathways should be taken into consideration during the therapeutic design for IDO1 inhibitors.

## The Niacin/Butyrate Receptor, GPR109A, Suppresses Inflammatory Responses in Myeloid Cells and the Colonic Epithelium

GPR109A (also known as the Hcar2) is a G-protein-coupled receptor (GPCR) that binds niacin (vitamin B3) and butyrate (64). Its first ligand, niacin, can be consumed from meats (e.g., fish, chicken, liver, turkey, and pork), vegetables (e.g., peas and mushrooms), cereal, and peanuts (65). Interestingly, some of the dietary requirement for niacin is provided by the production of nicotinic acid from the tryptophan/kynurenine pathway (66) mediated by IDO1. Its second ligand, butyrate, is produced by the gut microbiota *via* the fermentation of dietary fiber from plant products (67, 68).

Given the complexity of GPCR pathways (69), GPR109A plays variegated functions in different contexts. For example, GPR109A mediates the antidyslipidemic effect of nicotinic acid in decreasing low-density lipoprotein and triglycerides, and increasing high-density lipoprotein levels (70). This led to the use of nicotinic acid as an antihyperlipidemic agent (70), although its use is limited by the unfavorable side effect of cutaneous vasodilation, leading to skin flushing (70). This effect on the skin arises because, in addition to GPR109A expression by adipocytes (71), the receptor is also expressed by cutaneous immune cells (72) and epidermal Langerhan cells (73). Overall, GPR109A is generally highly expressed by myeloid cells such as neutrophils (74, 75), macrophages, monocytes, and dendritic cells (76, 77). It is also expressed by intestinal epithelial cells (78). Hence, it is not surprising for GPR109A to play an important role in regulating intestinal immunopathology and carcinogenesis.

The role of GPR109A has recently been described in suppressing colonic inflammation and cancer (77) (Figure 1C). In the latter study, the authors described GPR109A-deficient intestinal myeloid cells to express lower levels of IL-10 and exhibit a deficiency in their ability to stimulate Treg differentiation (77). The authors also showed that GPR109A^−/−^ colonic epithelial cells were unable to produce IL-18 (77). Finally, the authors showed that GPR109A deficiency exacerbated tumor development in the AOM/DSS model (77). Bone marrow transplant experiments revealed that the antitumor GPR109A effect was mediated by both the epithelial and immune compartments (77). Interestingly, treatment with niacin suppressed colonic inflammation and carcinogenesis in this model (77). In addition, similar findings were observed in another study that utilized high- versus low-fiber diets and GPR109A-deficient mice (79). In conclusion, GPR109A is a metabolic receptor for niacin and butyrate for which the therapeutic value, in GI inflammation and carcinogenesis, should be evaluated. Since GPR109A is highly expressed in myeloid cells, it would be additionally interesting to evaluate the role of this receptor in MDSCs. This is especially important given the MDSC role in suppressing CD8^+^ T cell antitumor immunity.

## The Itaconic Acid-Producing Enzyme, Irg1, in Regulating Succinate Levels and the Inflammatory Response in Macrophages

Immunoresponsive gene 1 (Irg1) is a mitochondria-associated metabolic enzyme that exhibits (1) an anti-inflammatory activity in host myeloid cells (80), and (2) an antimicrobial activity against pathogens (81, 82). Irg1 functions by decarboxylating *cis*-aconitic acid to produce itaconate, as part of the tricarboxylic acid (TCA) cycle (also known as the citric acid cycle) (Figure 1D) (81). The production of itaconate can generate an antimicrobial response by inhibiting the bacterial enzyme isocitrate lyase (81, 82). Moreover, in host myeloid cells, Irg1 functions by inhibiting succinate dehydrogenase (Sdh) (83–85), an enzyme that oxidizes succinic acid to fumaric acid within the TCA cycle. The inhibition of Sdh by Irg1-mediated itaconate sustains an accumulation of succinate during LPS stimulation and a decrease in the levels of fumarate and malate (80) (Figure 1D). Moreover, Irg1 suppresses inflammatory cytokine expression in myeloid cells such as IL-1β, IL-12, IL-6, IL-18, and the hypoxia-inducible factor 1 alpha (HIF-1α) (80). Hence, the mechanism of Irg1 and itaconate appears to be mediated by succinate inhibition, the latter of which can regulate major inflammatory pathways [such as HIF-1α (80)] in myeloid cells (86). In comparison to the previously described function of Irg1 against bacterial infections (81, 82), a recent study showed that the treatment with dimethyl itaconate did not affect the number of intracellular bacteria following *Salmonella typhimurium* infection of bone marrow-derived macrophages (80). Therefore, despite the established effect of Irg1 in myeloid cell responses (80), the antimicrobial effect of Irg1 remains debatable.

Given the above-described functions of Irg1, this molecule now represents an attractive target to investigate in GI diseases and cancer. To date, there have been no mechanistic reports on the function of Irg1 in the GI tract. However, two studies reported the induction of Irg1 in response to *Salmonella* infection in the chicken cecum. Hence, Irg1 represents a ripe topic for investigation in GI diseases and cancer (87–89).

## The Sphingosine-1-Phosphate Sphingolipid in Regulating Egress of Memory T Cells

Sphingosine-1-phosphate (S1P) is a signaling sphingolipid that regulates lymphocyte egress from secondary lymphoid organs (such as lymph nodes) (90) or non-lymphoid tissue (91, 92) (Figure 1E). S1P levels are high in efferent lymphatic ducts (higher than interstitial fluid of secondary lymphoid organs) (93), which attract lymphocytes that express high levels of sphingosine-1-phosphate receptor 1 (S1pr1) to egress out of the organs into the lymphatic ducts (93). This leads to recirculation of these lymphocytes *via* the lymphatic ducts and/or blood vessels (94). Once these lymphocytes egress, they downregulate their S1pr1 expression (95). Moreover, in the case of lymphocytes that constantly reside in non-lymphoid organs, such as tissue-resident memory T (T_RM_) cells, the consistent low expression of S1pr1 is necessary to sustain these cells within the organ without recirculation (92). In discussing the role of S1P, it is important to consider its dietary sources and the effect of its intake on GI disease outcome.

S1P is derived from dietary sphingolipids (96) that can impact the outcome of GI disease and cancer (97). Sphingosine-1-phosphate (S1P) lyase is an enzyme that degrades S1P in enterocytes, and its intestinal deletion leads to S1P accumulation in the colon, and an increase in T cells and colon carcinogenesis (97). The dietary source of S1P is variable and high levels can be consumed from dairy products, meats, and eggs, whereas vegetables and fruits contain lower levels (98). However, it is unclear whether the amount of dietary intake of S1P is critical. This is because lymphocyte differentiation and homing can be regulated at the level of expression of the S1P receptor, S1pr1, in lymphocytes—as will be reviewed in the following paragraph.

S1pr1 regulates T cell homing, which directly impacts T cell memory differentiation (92, 99–101). There are three types of memory T cells, which include (i) central memory T cells (T_CM_), (ii) effector memory T cells (T_EM_), and (iii) T_RM_. T_CM_ cells patrol lymph nodes and the white pulp of the spleen, and they express lymph node homing receptors (99–101). T_EM_ circulate between the blood and non-lymphoid tissue, but do not express homing receptors (99–101). T_RM_ are resident in the tissue and do not recirculate into the blood or secondary lymphoid organs (99–101). The homing of these cells is directly intertwined with their function and differentiation (99–101). As different types of memory T cells can exhibit different functions (99–101), the role of S1P and S1pr1 becomes critical in regulating disease outcome. This is especially important in regulating CD8^+^ T cell memory phenotypes to modulate their cytotoxic activities against tumors, or pro-inflammatory activities in autoimmune diseases. In support of the latter point, current drugs against S1pr1 in colon cancer have been considered (102) and are currently undergoing clinical trials for inflammatory bowel diseases and colorectal cancers.

## The Vitamin A Metabolite, atRA, in Regulating Epithelial MHC-I Expression and CD8^+^ T Cell Activation

All-trans-retinoic acid is a vitamin A metabolite that induces epithelial MHC-I expression in mouse models of colon carcinogenesis, therefore, triggering CD8^+^ T cell antitumor immunity (103). Vitamin A can occur in two basic forms, which are, respectively, obtained from two different dietary sources: (i) retinoids are supplied by animal food, whereas (ii) carotenoids are obtained from plant products (104). Retinyl palmitate constitutes the major form of vitamin A from animal food, which is converted in the small intestine to retinol, then to retinaldehyde, and then to retinoic acid (104). The synthesis of retinoic acid in the small intestine is mediated by retinaldehyde dehydrogenase enzymes, such as Aldh1a1, Aldh1a2, and Aldh1a3 (105–107), which are expressed by both intestinal epithelial and immune cells (103). In contrast to animal food sources, plant products provide carotenoids (provitamin A), which can be broken down into beta-carotene and then retinol in the intestinal mucosa (106). In both scenarios, the metabolic enzymes that regulate the synthesis or breakdown of retinoic acid in the small intestine provide an important checkpoint that can determine the resulting abundance of intestinal retinoic acid. The recent study by Bhattacharya et al. (103) demonstrated that in the AOM/DSS model, colon carcinogenesis led to downregulation of the atRA-synthesizing enzyme Aldh1a1. The downregulation of this enzyme was observed in both the epithelial and immune cell compartments (103). This was accompanied by an upregulation of the atRA-catabolizing enzyme, Cyp26A1, in the epithelial compartment of the colon mucosa (103). The authors went on to show that atRA reduced tumor burden in the AOM/DSS model and that this was mediated by the stimulation of atRA for epithelial MHC-I expression. Overall, the authors showed that mucosal inflammation, triggered by the host microbiota during colitis-associated colorectal cancer, stimulated atRA metabolism to decrease its levels, leading to reduced MHC-I and CD8^+^ activation (103). It will additionally be insightful to determine the effect of retinoic acid on CD8^+^ T cell memory differentiation as will be discussed in the following paragraph.

Previous studies have shown that atRA inhibits TGF-β-mediated Th17 differentiation and stimulates Treg differentiation (108–110). However, those studies also showed that atRA stimulates gut homing receptor expression, of α4β7 integrin and CD103, on T cells (108, 109). Hence, given the role of TGF-β-mediated induction of α4β7 integrin and CD103 in T_RM_ generation (111), it will be insightful to determine the mechanism in which atRA regulates T_RM_, T_CM_, and T_EM_ differentiation. Such analyses will also unravel novel information about the distinct roles of these memory T cell subtypes in colon carcinogenesis.

## Concluding Remarks

This article presents a number of scenarios in which metabolites and their receptors regulate the outcome of GI disease and cancer (Figure 1). In considering these scenarios, it is important to realize that several of these pathways can be triggered coincidentally within the same context of disease. Therefore, an integrated approach and combinatorial analyses are necessary to devise new therapeutic strategies that can synergistically improve prognoses. Theoretically, modulating these metabolic pathways to improve disease outcome should be feasible. However, what is not known are the “recipes” in which interrupting or altering these pathways would concomitantly lead to significantly improved outcomes.

## Author Contributions

Both ME-Z and JK contributed equally to literature review and concept development in the preparation of this review article.

## Conflict of Interest Statement

The authors declare that this review article was written in the absence of any commercial or financial relationships that could be construed as a potential conflict of interest.
